# Fall-related ocular trauma in patients over 90 years in tertiary ophthalmic center in Germany: 90-TOSG Report 1

**DOI:** 10.1007/s00417-023-06202-1

**Published:** 2023-08-17

**Authors:** Ayşe Güzin Taşlıpınar Uzel, Mariya Gosheva, Jonas Neubauer, Lydia Stock, Karl-Ulrich Bartz-Schmidt, Faik Gelisken, for the 90-Tübingen Ophthalmology Study Group, Karl-Ulrich Bartz-Schmidt, Spyridon Dimopoulos, Faik Gelisken, Mariya Gosheva, Jonas Neubauer, Lydia Stock, Lisa Vanessa Strudel, Jens Martin Rohrbach, Ayşe Güzin Taşlıpınar Uzel, Focke Ziemssen

**Affiliations:** 1 Department of Ophthalmology, Ufuk University, Ankara, Turkey; 2 Department of Ophthalmology, Eberhard Karls University, Tübingen, Baden-Württemberg Germany

**Keywords:** Fall, Ocular trauma, Open globe rupture, 90-years

## Abstract

**Purpose:**

To investigate the clinical characteristics of fall-related ocular trauma in patients over 90 years of age.

**Methods:**

Retrospective, medical record reviews. Patients over the age of 90 years treated in a tertiary center with fall-related ocular trauma were included in the study.

**Results:**

Fifty consecutive patients (fifty eyes) were analyzed. The mean age was 93.6 ± 1.8 years and 41 patients (82%) were female. The most common site of the injuries was orbital fracture (18 patients, 36%), accompanied with open globe rupture (OGR) in three patients, and globe contusion in two patients. Seventeen patients (34%) presented with OGR. Ocular trauma score in those patients was category 1 in 10 patients (58.8%) and category 2 in the others. Conjunctival hemorrhage and/or periocular contusion was seen in 14 patients (28%) and globe contusion in six patients (12%). At the presentation, the mean best corrected visual acuity (BCVA) was 2.82 ± 0.24 logMAR in patients with OGR and 1.98 ± 0.81 logMAR in six patients with globe contusion. Three of the patients with OGR had a final vision of 20/200 or better whereas the remaining patients had hand movements or less. The most common risk factors were female gender (82%) and use of antihypertensive drugs (46%).

**Conclusion:**

Patients with OGR had a poor visual outcome despite the early treatment. It is important to raise public awareness about of the poor prognosis of ocular injuries due to falls in the elderly population in order to establish preventive measures.


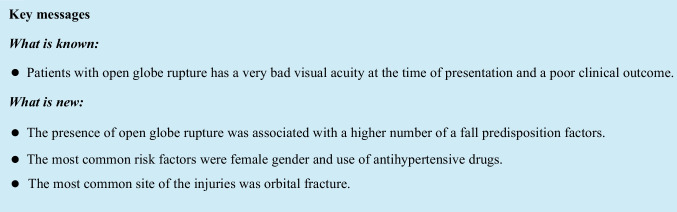


## Introduction

In western industrialized countries, the proportion of the elderly population is increasing with higher life expectancy. Consequently, new problems associated with older age have emarged. It is well-known that older age is an independent risk factor for falls, with one in two people over the age of 80 years reporting a fall once a year [1, 2].

The annual incidence of eye injuries in patients over 65 years of age is 12 per 100.000 [3]. Falls are the most common cause of eye injury in this population [4]. The frequency of eye trauma in falls and vision-threatening complications increases also with older age and the characteristics of eye injuries in the elderly are different from those in the younger population [2, 5]. On the other hand, the inconsistency in definition of the elderly population in various reports makes an analysis difficult. Some studies included patients in the sixth and seventh decades of life, who may still be in an active working life and not reflecting the typical characteristics about the type and features of eye injuries in very old patients. It is likely that trauma will have different outcomes in very elderly patients who have more comorbidities and a relatively sedentary lifestyle with a low level of physical activity.

The aim of this study is to investigate the demographic and clinical characteristics of patients over 90 years of age presenting with fall-related ocular trauma at a German tertiary eye clinic.

## Methods

In this retrospective study, the medical records of all patients over the age of 90 years, who presented with a new eye trauma between 2003 and 2021 at the Department of Ophthalmology of the University of Tübingen, were reviewed and fall-related cases were included in the analysis. Approval was obtained from the institutional ethics committee and this study was conducted in accordance with the Declaration of Helsinki.

Patients admitted primarily to the Ophthalmology Department of the Eberhard Karls University or referred from other departments were identified using the electronic chart archive.

The demographics of the patients, clinical characteristics of the eyes, visual outcome, treatment modalities and the risk factors associated with falls were analyzed [2].

All patients underwent a complete ophthalmological and a systemic examination was performed if necessary. Best corrected visual acuity (BCVA) was measured using Snellen charts and converted to the logarithm of the minimum angle of resolution (logMAR) for further analysis. Intraocular pressures were evaluated with an applanation tonometer if necessary. If an orbital fracture or extraorbital involvement was suspected, computed tomography or further examinations were performed. The ICD-10 German modification and Birmingham eye trauma classifications were used [6]. The injury zone was classified as follows: zone 1 includes involvement of the cornea or limbus, zone 2 includes the area within 5 mm from the limbus and zone 3 if the injured area extended more than 5 mm from the limbus. The Ocular trauma score (OTS) was also calculated [7].

IBM SPSS Statistics 21 was used for statistical analysis. The normality of the data was tested using the Shapiro–Wilk test. Dependent variables were analyzed using a paired samples t-test. Logistic regression analysis was performed to assess association between risk factors for fall, presence of previous surgery and the development of open globe rupture (OGR). Statistical significance was accepted as a p-value of 0.05 or less.

## Results

A total of 52 consecutive patients over the age of 90 years, who presented with ocular trauma were identified. Two patients without a history of fall were excluded. The data of 50 patients (50 eyes) was analyzed. The mean age was 93.6 ± 1,8 years. 41 patients (82%) were female. In half of the patients, the right eye was affected. Hospitalization rate was 42%, with a mean of 7.4 ± 3.9 days.

The primary diagnosis was conjunctival hemorrhage or periocular contusions in 28% (14/50) of patients. Globe contusion including hyphema and vitreous hemorrhage was detected in 12% (6/50) of the patients. In total, 34% (17/50) of patients had OGR. Orbital fracture was present in 36% (18/50) of the patients, 11.1% (2/18) of them with orbital contusion, 16.6% (3/18) with OGR, and 11.1% (2/18) with globe contusion. Orbital floor fractures accounted for 94.4% (17/18) of all orbital fractures. Facial bone fractures were also present in seven patients. One patient had severe lid injury including lacrimal sac and canaliculus laceration and another one conjunctival laceration. In addition, thumb fracture in one patient and rib fracture in another patient were diagnosed.

At the presentation, the mean BCVA in the study eyes of the total 45 patients with available data was 1.68 ± 1.2 logMAR. The mean baseline BCVAs were 2.82 ± 0.24 logMAR in 17 patients with OGR, 0.76 ± 0.90 logMAR in 13 patients with only periocular contusion, 0.78 ± 0.86 logMAR in 10 patients with only orbital fracture, and 1.98 ± 0.81 logMAR in five patients with globe contusion. In patients with OGR, the initial vision was 3.0 logMAR (no light perception) in 58.8% of the patients and 2.7 logMAR (light perception) in 29.4% of the patients. The mean BCVA of fellow eyes (39 patients with available data) was 0.78 ± 0.77 logMAR. In 25.6% of the patients the mean BCVA was 1.0 logMAR or less. Information about the visual acuity prior to presentation was available in 2 cases and in both was no better than hand movement.

Information regarding the place of the fall was available for 29 of 50 patients (58%). Most of these patients (86.2%) experienced an indoor fall. 10 patients fell in a nursing home and 12 patients fell at home.

Of the 17 eyes of patients with OGR, 29.4% (5/17) of had scleral rupture, 23.5% (4/17) had corneal rupture, and 47.1% (8/17) had corneoscleral rupture. Rupture in zone 1 was found in 64.7% (11/17), in zone 2 in 29.4% (5/17) and in zone 3 in 5.9% (1/17) of the patients. In 47.1% (8/17) of the patients, dehiscence occurred at the incision of the previous cataract surgery. Fundus examination could not be performed in 88.2% (15/17) of the patients due to the severity of the injury and/or the accompanying hyphema. While 64.7% (11/17) of the patients had vitreous hemorrhage, 58.8% (10/17) had hyphema, and 5.9% (1/17) had choroidal detachment. Intraocular tissue prolapse was found in 94.1% (16/17) of the patients, 56.2% of them with choroidal and retinal tissue. The ocular trauma score in patients with OGR was category 1 in 10 patients (58.8%) and category 2 in the others. In our population the BCVA in most of the patients in these categories 1 or 2, was 2.3 logMAR (hand movement) or worse.

Four patients (22.2%) with orbital fractures were operated by plastic surgery or otorhinolaryngology departments. Lacrimal sac and canalicular repair with silicone tube intubation was performed in one patient. All 17 patients with OGR underwent their primary surgical intervention within 24 h after admission. Primary evisceration was performed in 11.8% (2/17). Systemic antibiotics were administrated if needed. Thirteen patients (76.4%) were operated under general anesthesia. The mean operation duration was 61.6 ± 29.1 min. Vitrectomy was performed in 35.2% (6/17) of the patients as a secondary intervention. The mean hospital stay was 8 ± 3.7 days (range 2–16 days), and the mean follow-up time 4.7 ± 10.7 months (range 0–33.7 months). Nine patients (52.9%) had at least one-month follow-up. In these patients with available follow-up, the BCVA was 2.75 ± 0.22 logMAR (20/11000) and 1.97 ± 1.05 logMAR (20/2000) at baseline and at last visit, respectively (*p* = 0.028). In total, three of these patients had a final vision better than 20/200. During the follow-up, one eye developed phthisis and another eye corneal decompensation. Endophthalmitis was not noted during the follow-up.

The main accompanying systemic diseases were arterial hypertension in 46% followed by cognitive impairment with 42% of the patients. The most common risk factors for fall were female gender (82%) and use of antihypertensive drugs (46%). The mean number of risk factors in all patients was 1.8 ± 0.8. (Tables 1 and 2) The increased number of risk factors was associated with OGR (OR 3.17 [95% CI, 1.30–7.71], *p* = 0.011). Of the patients, 28.8% (13/45) had no previous ocular surgery, 66,6% (30/45) had previously undergone phacoemulsification, and 4% (2/45) had previously undergone keratoplasty. No information was available in the records of five patients. No relationship between the presence of previous surgery, cognitive impairment or any risk factor and the occurrence of OGR was found. The most common risk factors in the patients presenting with OGR were female gender (88.2%, 15/17) and use of antihypertensive drugs (41.1%, 7/17), followed by visual impairment (23.5%, 4/17) and physical disability (23.5%, 4/17). Recurrent fall history was recorded in 11.7% (2/17) of the patients with OGR, incontinence 11.7% (2/17), Parkinson disease 5.8% (1/17), depression 5.8% (1/17), vertigo 5.8% (1/17) and walking aid use 5.8% (1/17).

**Table 1:** Risk Factors for Fall-related Ocular Trauma

	*n* (total 50 patients) (%)
Female gender	41 (82%)
Use of antihypertensive medications	23 (46%)
Visual impairment*	10 (20%)
Physical disability	4 (8%)
Parkinson disease	3 (6%)
Recurrent fall history	3 (6%)
Incontinence	3 (6%)
Depression	1 (2%)
Vertigo	1 (2%)
Walking aid use	1 (2%)

*If best corrected visual acuity of fellow eye was ≤ 20/200, it was accepted as visual impairment

**Table 2:** Relationship Between Types of Injuries and Number of Risk Factors

Number of the Risk Factors* (n, %)						
Injury Type	n	No	One	Two	Three	Four
Severe Globe Contusion	6	2 (33.3%)	2 (33.3%)	1 (16.6%)	0	1 (16.6%)
Open Globe Rupture	17	3 (17.6%)	7 (41.1%)	3 (17.6%)	3 (17.6%)	1 (5.8%)
Only Orbital Fracture	13	6 (46.1%)	3 (23%)	3 (23%)	1 (7.6%)	0
Only Periocular Contusion or Mild Globe Contusion	14	5 (35.7%)	5 (35.7%)	3 (21.42%)	0	1 (7.1%)

*Risk factors: female gender, use of antihypertensive medications, visual impairment, physical

disability, Parkinson disease, recurrent fall history, incontinence, depression, vertigo and walking aid

use

## Discussion

In the presented study, orbital fracture and OGR were found to be most common types of eye injuries due to fall in patients over the age of 90 years, who admitted into a tertiary eye center in Germany. The OGR was seen mostly in female patients and patients with arterial hypertension. In the most of the patients with OGR, BCVA at presentation was light perception or worse. OGR was often accompanied with prolapse of the intraocular tissue and poor visual outcome.

Ocular trauma is seen generally more common in men, when all age groups, gender and all etiological causes are considered [8]. However, fall-related eye trauma is more common in women [3, 9, 10]. In studies conducted on patients over the age of 65 years, women comprise 60–65% of the patients with open globe injuries due to falls [3, 5]. In the present study, 82% of the patients were female. Possible reasons for the female dominancy are higher life expectancy than man, and the effect of the Second World War on the demographics [8].

In a previous study reporting the fall-related ocular injuries, the hospitalization rate was reported to be 91% and the need for intensive care was 8.5% [11]. Only 42% of the patients in the present study were hospitalized, and none of them required intensive care. These discrepancies could most likely be caused by the inclusion of different of the age groups and different types of falls (such as falls from height) in the other study cohorts. Falls leading to ocular trauma seen in very old patients may be low-impact falls with mostly local effects.

The frequency of orbital floor fractures due to fall has been reported to be 16% with an increase in older age [3]. In falls over 61 years of age, orbital fracture was seen 21% of the patients and none of the patients had permanent visual or functional impairment due to the fracture [12]. In another study in patients over 65 years, orbital fractures were with 57% the most common type of injury in fall-related ocular trauma [11]. Orbital revision was performed in 13% of these patients. In the same study, the high rate of hospitalization and need for intensive care suggests that the falls in the study were high-impact falls, such as falling from a height. In the present study, orbital fracture was seen in 36% of the patients and 22% of these patients required surgery. Almost all orbital fractures were floor fractures as seen usually in blunt type of trauma [13].

In line with the other reports, eyes were mostly ruptured in zones 1 and 2, and almost half of these eyes presented with a dehiscence at the previous surgical incision of the cataract surgery [4]. A previous cataract surgery was reported in 39% of patients with OGR over the age of 65 [14]. In the present study, although this rate was quite high (14 of 17 patients, 82.3%), no relationship was found between the injury and the incision side of the previous surgery. The reason for this difference may be the change in cataract surgery technique over the years, namely the tunnel incision technique for phacoemulsification versus the larger corneoscleral incision for the extracapsular cataract surgery and the different representation of these groups in previous studies. It has been reported that the tunnel technique provides stronger wound tissue than corneal or corneoscleral incisions [14].

9% of patients over the 70 years of age with fall-related OGR had no light perception at presentation [5]. This rate has been reported to be 24% in all types of open globe trauma [4]. Another study reported a visual acuity of light perception or worse in 61% of such patients [11]. In our study, 59% of patients had no light perception and 88.2% had light perception or worse. We also noted the hand movement was the most favorable BCVA in patients with OGR at presentation. Although there was a significant increase in BCVA at the last visit, only three patients with OGR had a vision better than 20/200.

The rate of at least one-month follow-up examination was low (53%). Most patients with OGR who were not followed up had no light perception at discharge. In a study investigating open globe injuries in patients over the age of 70 of age, the rate of primary evisceration rate was 4%, and increased to 9% during the follow-up [4]. In the presented study, two patients (12%) underwent a primary evisceration with severe intraocular tissue prolapse at the presentation. Almost half of the patients in our cohort had prolapse of intraocular tissue including uvea and retina. However, no further evisceration was performed during the follow-up. Vitrectomy was required in 35% of the patients after the primary repair, which is consistent with a previous report [5].

Risk factors such as cognitive impairment, use of antihypertensive drugs and the number of chronic diseases have been found to be associated with more serious injuries due to fall [15, 16]. In our study, cognitive impairment was found in 53% of patients with OGR, 36% of the patients without OGR in our study. Cognitive impairment is an important feature limiting protective responses due to the lack of defense reflexes during the fall. We could not find a relationship between cognitive impairment and OGR, possible because of absence of detailed medical history and the small number of patients in our study cohort. However, even without severe cognitive impairment, reduced muscle strength, and age-related physical performance of the patients over the age of 90 years of age may limit the defense during fall. The increased number of fall predisposition factors was also associated with the presence of OGR. Previous reports in middle-aged patients found OGR after fall-related ocular trauma in 9–12%, whereas in our study it was found in 34% of patients over 90 years of age [11, 12]. Increasing comorbidities with older age are high likely the reason for the higher frequency of severe type of the trauma.

The limitations of the study are the small number of patients with limited follow-up, which is related to the very old age of the cohort. The retrospective design also limited the access to detailed psychological and sociodemographic risk factors for fall. We could not use standardized questionnaire. Consequently, environmental related factors such as living alone, a family living or living with professional health care staff, could not be analyzed. In the evaluation of the visual acuity, the effect of the existing age-related diseases, such as macular degeneration or glaucoma could not be analyzed in detail.

In conclusion, orbital fracture and OGR are the most common eye injuries after falls in patients over the 90 years of age. OGR leads to poor visual outcome, despite the maximal surgical repair. The presented study underlines the importance of multidisciplinary preventive programs for fall-related eye injuries in the elderly population.
